# Supranutritional selenium level minimizes high concentrate diet-induced epithelial injury by alleviating oxidative stress and apoptosis in colon of goat

**DOI:** 10.1186/s12917-020-02653-4

**Published:** 2020-11-27

**Authors:** Saba Parveen Samo, Moolchand Malhi, Allah Bux Kachiwal, Javaid Ali Gadahi, Fahmida Parveen, Nazeer Hussain Kalhoro, Yan Lei

**Affiliations:** 1 Department Veterinary Physiology and Biochemistry, Sindh Agricultural University, 70060 Tando Jam, Pakistan; 2 Department of Veterinary Parasitology, Sindh Agricultural University, 70060 Tandojam, Pakistan; 3 Department of Veterinary Pathology, Sindh Agricultural University, 70060 Tandojam, Pakistan; 4 Sindh Poultry Vaccine Centre, Animal Science Complex, Korangi , 74900 Karachi, Pakistan; 5 Dairy Herd Improvement Center, Henan Animal Husbandry Bureau, 450046 Zhengzhou, China

**Keywords:** selenium, high concentrate diet, oxidative stress, apoptosis, goat colon

## Abstract

**Background:**

High concentrate (HC) diet-induced oxidative stress causes gut epithelial damages associated with apoptosis. Selenium (Se) being an integral component of glutathione peroxidase (GSH-Px) plays an important role in antioxidant defense system. Therefore, increasing dietary Se level would alleviate HC diet-induced injuries in gut mucosa. The present study investigated eighteen cross-bred goats, randomly divided into three groups (n = 6/group) fed either low concentrate (LC, roughage: concentrate ratio 65:35), high concentrate (HC, 35:65) or HC plus Se (HC-SY) diets for 10 weeks. Se was supplemented at the dose rate of 0.5 mg Se kg^− 1^ diet in the form of selenium yeast. The background Se level in HC and LC diets were 0.15 and 0.035 mg.kg^− 1^ diet, respectively. The Se at the dose of 0.115 mg.kg^− 1^ diet was added in LC diet to make its concentration equivalent to HC diet and with the supplementation of 0.5 mg Se kg^− 1^, the goats in group HC-SY received total Se by 0.65 mg.kg^− 1^ diet.

**Results:**

The molar concentrations of individual and total short chain fatty acids (TSCFA) significantly increased (*P* < 0.05) with simultaneous decrease in pH of colonic fluid in goats of HC and HC-SY groups compared with LC goats. HC diet induced loss of epithelial integrity, inflammation and loss of goblet cells in colonic mucosa associated with higher lipopolysaccharide (LPS) concentrations in colonic fluid whereas, the addition of SY in HC diet alleviated such damaging changes. Compared with LC, the HC diet elevated malondialdehyde (MDA) level with concurrent decrease in GSH-Px and superoxide dismutase (SOD) activities, while SY supplementation attenuated these changes and improved antioxidant status in colonic epithelium. Moreover, epithelial injury and oxidative stress in colon of HC goats were associated with increased apoptosis as evidenced by downregulation of bcl2 and upregulation of bax, caspases 3 and 8 mRNA expressions compared with LC goats. On contrary, addition of SY in HC (HC-SY) diet alleviated these changes by modulating expression of apoptotic genes in colonic epithelium.

**Conclusions:**

Our data suggest that supranutritional level of Se attenuates HC diet-induced oxidative stress and apoptosis and thereby minimizes the epithelial injury in colon of goats.

## Background

High concentrate (HC) diet is fed to animals in order to maximize the growth and production performances [1]. Long term feeding of HC diet results in digestive disturbances and leads to complex systemic problems. In ruminants, feeding HC diet is concerned with an abnormal increase in the fermentation capacity of rumen and hindgut which reduces the pH due to accumulation of organic acids [2, 3]. The low pH elicits a substantial release of bacterial endotoxins (Lipopolysaccharide, LPS) which alters the permeability of gut [4–6]. The gut epithelium acts as principle barrier against invading organisms like bacteria, toxins and various chemical agents, it also plays an active part in immune response and in the maintenance of nutrient absorption [7, 8]. In addition, being a single layered structure, the colonic epithelium is much more predisposed to abnormal luminal environment compared with multilayered ruminal epithelium [7]. Moreover, the existence of natural defensive mechanism to cope with increased acidity through microbial activities and buffering action from saliva in rumen is lacking in the large intestine. This condition makes the large intestine more prone to epithelial damage which causes LPS translocation and leads to systemic complications [9].

It has been shown that HC diet-induced colonic epithelial damage results from apoptosis which is associated with oxidative stress (OS). Tao et al. [10] and Ye et al. [3] reported the excessive formation and accumulation of reactive oxygen species (ROS) such as hydrogen peroxide (H_2_O_2_) and lipid peroxides and the reduced activities of antioxidant enzymes in colonic epithelium of goats. OS is a key mediator to activate the cell’s apoptotic signals [11, 12]. Recent findings have shown that HC diet induces mucosal injuries in cecum and colonic epithelia through apoptosis which is associated with increased oxidative stress. The imbalance in the expression of pro- and anti-apoptotic genes disturbs the bcl2/bax ratio and activates caspases which lead to cell death in colonic epithelial tissue of goats fed HC diet [13].

Selenium (Se), a well-known biologically vital trace element, is an integral part of glutathione peroxidase (GSH-Px) which makes an important part of anti-oxidation defense system in animal body [14, 15]. GSH-Px is usually found in almost all the vital tissues of ruminant’s body including liver, kidney, lungs, pancreas, spleen, skeletal muscles, reproductive organs and gastrointestinal tract [16–18]. The normal synthesis and secretion of tissue GSH-Px depends upon bio-availability of Se in the organ which in turn depends upon its concentration in the diet fed to animals. In addition, the passage rate down the digestive tract, absorption and retention of Se in tissues is higher in sheep fed HC diet compared with those fed high forage diet [19]. Moreover, the sheep and goats fed Se at supranutritional doses (20–30 times higher than the normal recommended dose) increased tissue Se retention and improved antioxidant stability without showing any toxic effects [20, 21]. Besides antioxidant activity, Se is well known for its role in cell proliferation, immune-modulation, inflammation and apoptosis. At excess doses (0.6 mg.kg^− 1^diet) dietary Se reduced DNA damage in prostate cells and peripheral lymphocytes in dogs [22] and reduced apoptosis of neutrophils in cows [23]. Several studies have examined the protective role of Se under various stressful conditions and it has been reported that dietary Se supplementation alleviated alflatoxin B1 (AFB_1_) induced injury of bursa of fabricius in chicken [24], *schistosoma mansoni*-induced hepatic injury in mice [25] and high fat diet-induced aortic injury in rabbit [26] through modulation of cell cycle and apoptotic genes.

Moreover, the role of Se as an anti-stressor has been evaluated in domestic animals. Dietary Se supplementation reduced oxidative stress and improved intestinal function in pigs exposed to heat stress [27]. Supplementation of Se at supranutritional level increased growth rate and improved muscle health in heat-stressed sheep [28–31]. Keeping in view the beneficial effects of extra dose of Se especially in stressful conditions, we speculated that increasing Se level in HC diet would alleviate the HC diet-induced colonic injury by improving antioxidative stability and inhibiting the apoptotic pathway in colonic epithelium. The present study was therefore designed to evaluate role of selenium yeast (SY) against HC diet induced fermentation pattern, morphological changes, the status of epithelial cellular oxidative stress and apoptosis in the colon of goat.

## Results

### Fermentation pattern and LPS level in colon

**Fig. 1:**
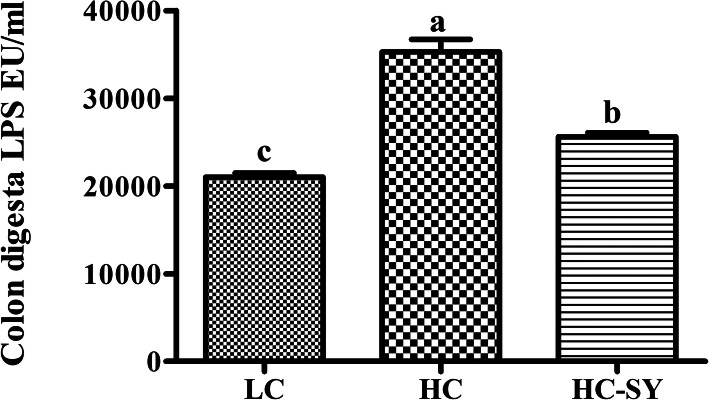
The Effects of LC, HC and HC-SY diets on LPS concentration in digesta of colon. Level of LPS was calculated as endotoxin unit per ml^− 1^ (EU/ml). Goats were fed experimental diets for 10 weeks with total 0.15, 0.15 and 0.65 mg.kg^− 1^ diet Se concentration in LC, HC and HC-SY respectively. Values are mean ± S.E and ^a, b, c^ different letters on the bars exhibit the differences between groups with *P* < 0.05

The molar concentrations of acetate, propionate, butyrate, total short chain fatty acids (TSCFA) and the acetate: propionate ratio significantly increased (P < 0.05) in colonic fluid of goats fed HC and HC-SY diets compared with those fed LC diet (Table 1). Simultaneously, the pH of colonic fluid significantly decreased (*P* < 0.05) in HC and HC-SY compared with LC goats. However, no significant differences (*P* > 0.05) were found in the SCFA concentration and pH between HC and HC-SY treatments. Lipopolysachharide (LPS) level increased (*P* < 0.05) by 67.81% and 21.87% in HC and HC-SY groups, respectively compared with LC (Fig. 1). However, the Se treatment significantly reduced (*P* < 0.05) the LPS level in HC-SY by 27.37% compared with HC.

**Table 1:** The effect of LC, HC and HC-SY diets on SCFA concentration and pH in colonic fluid of goat

Items	Treatments		
	**LC**	**HC**	**HC-SY**
Acetate (mmol)	44.50 ± 3.50^a^	62.50 ± 2.32^b^	59.25 ± 3.11^b^
Propionate (mmol)	21.00 ± 2.08^a^	32.75 ± 2.92^b^	31.15 ± 2.39^b^
Butyrate (mmol)	9.50 ± 0.95^a^	19.75 ± 1.70^b^	21.25 ± 1.37^b^
TSCFA (mmol)	55.68 ± 1.37^a^	92.29 ± 3.32^b^	89.25 ± 1.55^b^
Ac: Pr Ratio	1.97 ± 0.21^a^	2.85 ± 0.13^b^	2.45 ± 0.18^b^
pH	7.86. ± 0.12^a^	5.81 ± 0.11^b^	6.11 ± 0.12^b^

*Ac:Pr *acetate to propionate ratio, *TSCFA *total short chain fatty acid. Goats were fed low concentrate (LC), high concentrate (HC) and HC plus selenium (HC-SY) diets for a period of 10 weeks. Total Se concentrations in LC, HC and HC-SY diets were 0.15, 0.15 and 0.65 mg.kg^− 1^ diet, respectively. Values are means ± SEM. ^a, b, c^ and values with different superscripts were considered significant at *P* < 0.05

### Histo-morphology and damage score of epithelial tissue in the colon

**Fig. 2:**
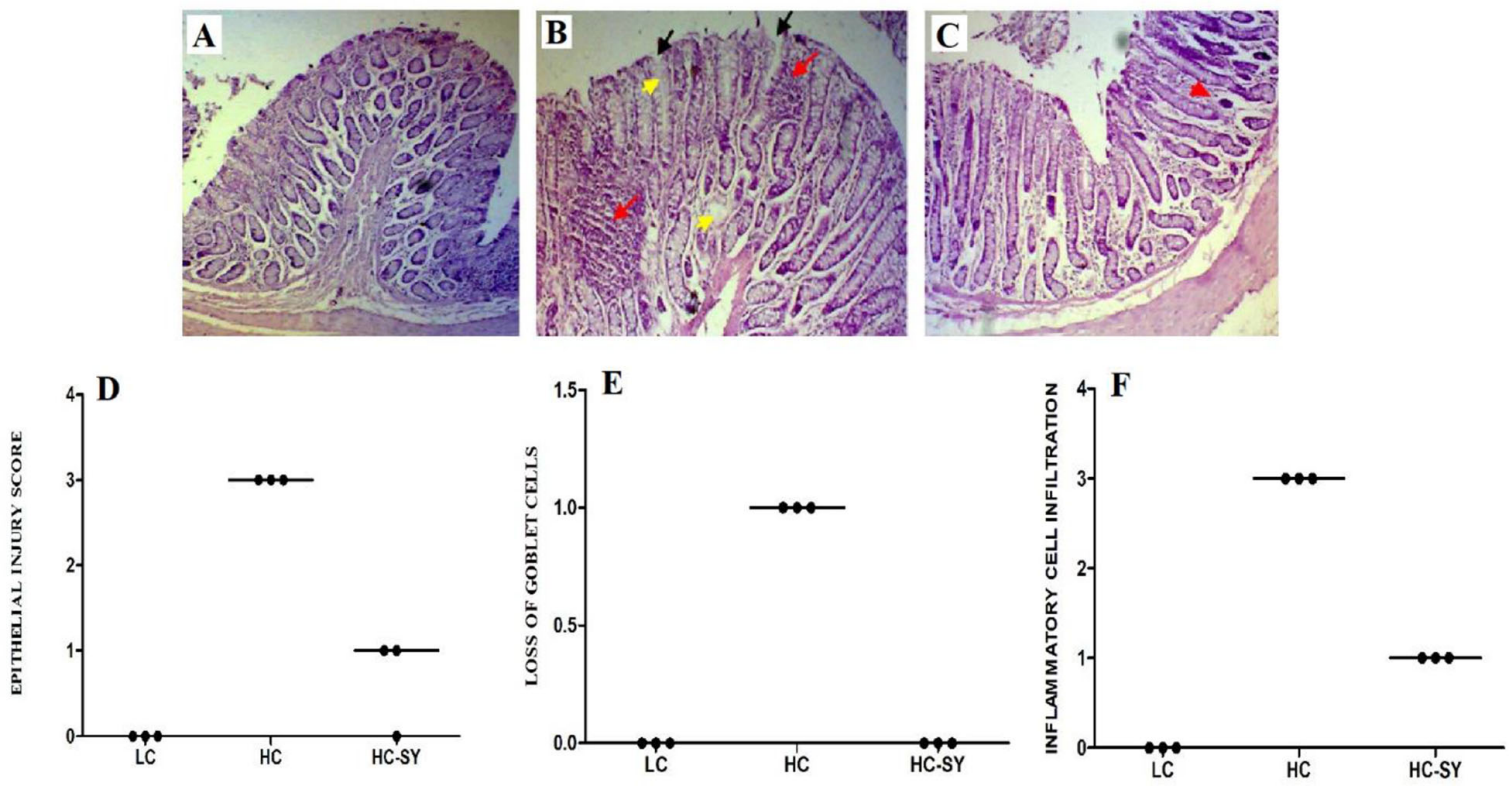
The comparison of histological damages in colonic epithelium of LC, HC and HC-SY. Goats were fed experimental diets for 10 weeks with total 0.15, 0.15 and 0.65 mg.kg^− 1^ diet Se concentration in LC, HC and HC-SY respectively. Colon (*n* = 6) from each group were processed for histological evaluation: colon section of LC group (**a**, scale bar = 100 µm); HC group (**b**, scale bar = 100 µm) and HC-SY (**c**, scale bar = 100 µm) at 10X magnification. Representative histological sections of the colon tissue were stained by H&E. Colon injury scoring was categorized on the basis of histological damages indicated by black, yellow and red arrow showing epithelial injury score (**d**), goblet cell depletion (**e**) and inflammatory cell infiltration (**f**) respectively. Values are means ± SEM. The significance was considered at *P* < 0.05

Effects of different dietary treatments on colonic histomorphology showed severe damages in colonic mucosa characterized by massive loss of epithelial integrity, depletion of goblet cells and infiltration of inflammatory cells in goats fed HC diet compared with those fed LC diet which showed normal epithelial structure (Fig. 2a and b). Whereas, HC-SY goats showed slight epithelial injury which suggests that Se supplementation reduced damaging effects of HC diet on colonic epithelium (Fig. 2c). Analysis of mucosal injury (Fig. 2d-f) showed that HC diet caused significantly higher (*P* < 0.05) injury score and inflammatory cell infiltration compared with LC diet. However, Se supplementation (HC-SY) significantly reduced (*P* < 0.05) injury score compared with HC diet.

### Markers of oxidative stress in colonic epithelium

**Fig. 3:**
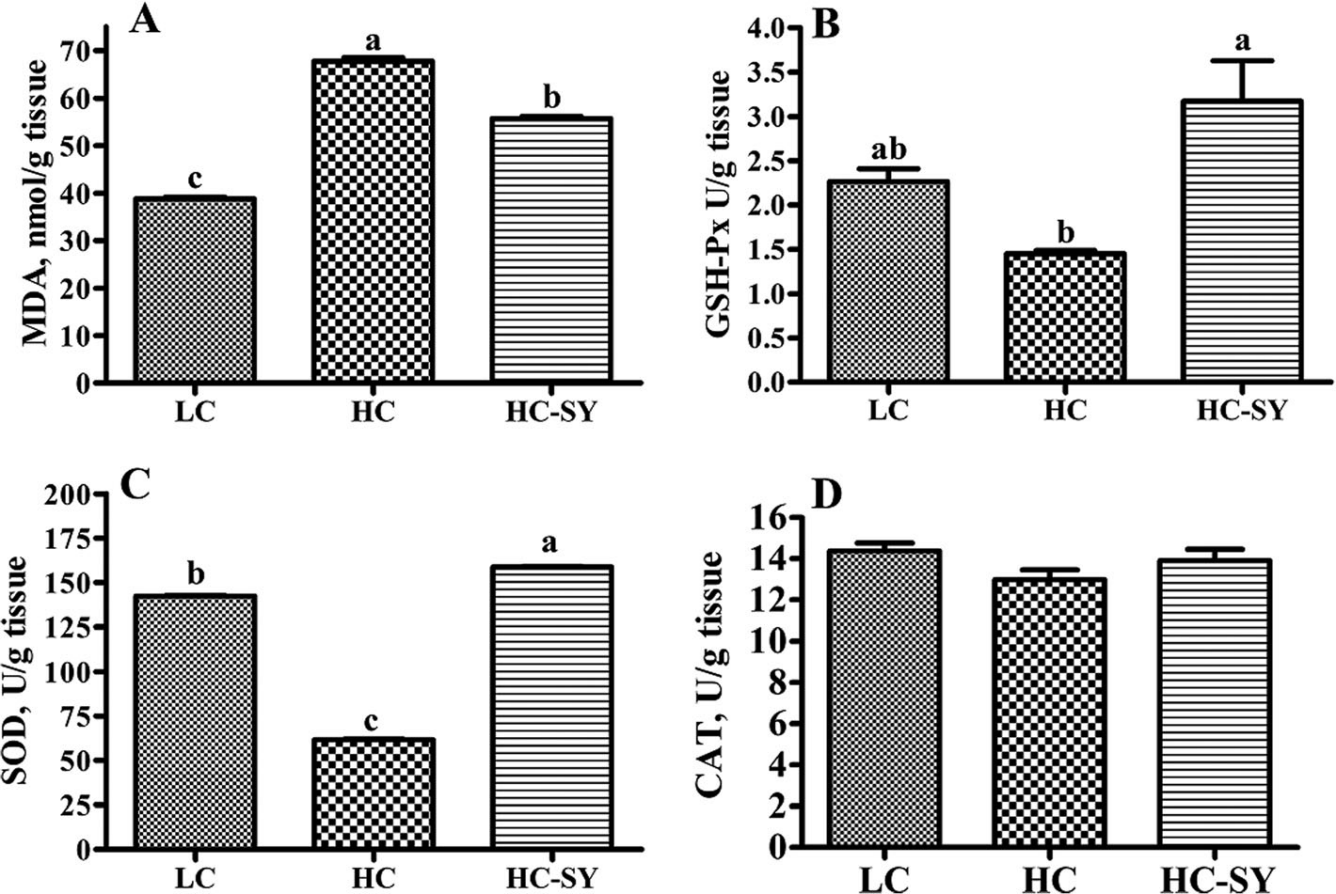
The effects of LC, HC and HC-SY diets on oxidative markers in colonic epithelium. Level of MDA (**a**), GSH-Px (**b**), SOD (**c**) and CAT (**d**) were determined in the goats fed experimental diets for 10 weeks with total 0.15, 0.15 and 0.65 mg.kg^− 1^ diet Se concentration in LC, HC and HC-SY respectively. The level of oxidative markers was calculated with OD values by using spectrophotometer. Values are mean ± S.E and ^a, b, c^ different letters on the bars exhibit the differences between groups with *P* < 0.05

The tissue melondialdehyde (MDA) level increased (*P* < 0.05) by 74.77% and 43.77% in colonic epithelium of HC and HC-SY goats, respectively compared with LC goats. Supplementation of Se in diet (HC-SY) reduced (*P* < 0.05) MDA level by 17.7% compared with HC diet (Fig. 3a). Though, the tissue glutathione peroxidase (GSH-Px) activity in colon was slightly lower in HC and slightly higher in HC-SY goats but statistically non-significant compared with LC goats. However, the GSH-Px activity increased (*P* < 0.05) by 120% in HC-SY compared with HC group (Fig. 3b). The tissue superoxide dismutase (SOD) activity in colon reduced (*P* < 0.05) by 56.7% in HC and increased (*P* < 0.05) by 11.56% in HC-SY compared with LC goats. However, the increase in SOD activity was 157.7% (*P* < 0.05) higher in HC-SY compared with HC goats (Fig. 3c). Nevertheless, tissue catalase (CAT) activity in colon did not show significant (*P* > 0.05) difference among the groups (Fig. 3d).

### Expression of apoptotic genes in colonic epithelium

**Fig. 4:**
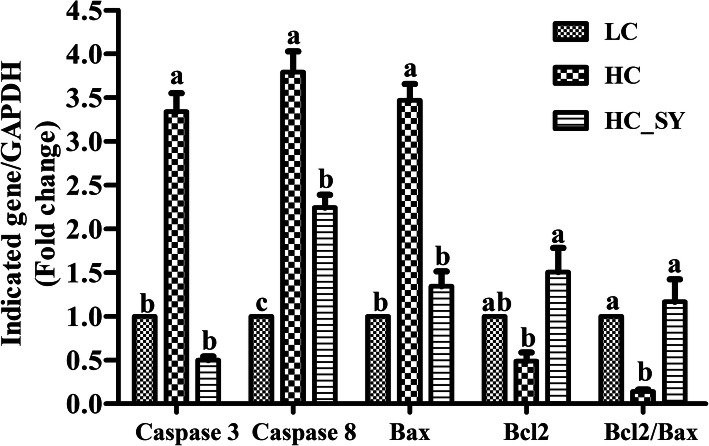
Effect of LC, HC and HC-SY diets on apoptosis related genes in colonic epithelium. Goats were fed experimental diets for 10 weeks with total 0.15, 0.15 and 0.65 mg.kg^− 1^ diet Se concentration in LC, HC and HC-SY respectively. The levels of gene expression were calculated with real-time PCR in comparison with GAPDH. Values are expressed as mean ± S.E and ^a, b, c^ different letters on the bars exhibit the differences between groups with *P* < 0.05

Figure 4 shows the mRNA expression level of apoptotic genes in colonic epithelium of goats. HC diet significantly increased (*P* < 0.05) the expression levels of bax (Pro-apoptotic) and caspase genes compared with LC and HC-SY diets, however, no significant difference (*P* > 0.05) was observed between LC and HC-SY fed goats. Bcl2 (Anti-apoptotic) expression significantly increased (*P* < 0.05) in colonic epithelium of HC-SY compared with HC goats; however, no significant difference (*P* > 0.05) was observed in bcl2 expression in LC compared with HC and HC-SY goats. HC diet significantly increased (*P* < 0.05) the caspase 3 expression level compared with LC and HC-SY diets. Though, the caspase 3 expression level was significantly higher (*P* < 0.05) in HC-SY compared with LC goats but it was significantly lower (*P* < 0.05) compared with HC goats. Bcl2/bax ratio significantly decreased (*P* < 0.05) in HC compared with LC and HC-SY goats, however, no significant difference (*P* > 0.05) was observed between LC and HC-SY goats.

## Discussion

### Fermentation pattern and LPS level in colon

In the current study, the molar concentrations of individual short chain fatty acid (SCFA) i.e. acetate, propionate and butyrate, and thus total SCFA increased in colonic fluid of goats fed high concentrate (HC) and HC plus Se (HC-SY) diets compared with those fed low concentrate (LC) diet. Concomitantly with increased total SCFA concentrations, the digesta pH decreased in colon of goats fed HC and HC-SY diets. Previous studies have reported that feeding HC diets enhanced fermentation rates and decreased digesta pH in colon of goats [3] sheep [32] and cattle [33]. Moreover, linear increase in SCFA concentrations and the decrease in pH with number of days fed HC diet suggests that long term feeding with HC diet severely increase the fermentation [32]. Nevertheless, colonic fermentation pattern in goats fed Se supplemented HC diet showed no significant difference compared with those fed HC diet without Se supplementation. The effects of dietary Se on fermentation pattern varies with dose and type of diet fed to ruminants. Cows fed Se (mg.kg^− 1^diet) at dose rates up to 0.3 increased ruminal SCFA concentrations whereas increasing dose up to 0.45 did not cause further increase [34]. At doses (0.3–0.9 mg.kg^− 1^ diet) Se did not alter fermentation pattern in lambs fed either 50% or 70% grain diet [20]. It seems that HC diet-induced increase in fermentation rate might have reached at a point of saturation which could have masked the Se effects.

Lipopolysaccharides (LPS) act as endotoxins and immunogens, and are the part of outer cell wall layer of gram negative bacteria. Increased LPS concentrations have been reported in ruminal fluid and hind gut (Cecal and colonic) digesta as well as in feces of ruminants fed HC diets [33, 35]. In the present study, HC diet elevated LPS concentration in colonic digesta. Metzler-Zebeli et al. [36] and Ye et al. [3] demonstrated an increase in LPS concentrations in colonic digesta of goats fed 60% and 65% grain rich diets, respectively. While passing through various growth phases, bacteria normally shed LPS and other endotoxins during the logarithmic phase; in addition they are also derived from disintegration and lysis of bacteria [33, 37]. It has been demonstrated that HC diet-induced reduction in digesta pH causes death and lysis of gram negative bacteria and eventually results in elevation and accumulation of LPS in gut lumen [33]. In the present study, the addition of Se in HC diet inhibited the increase in colonic LPS concentration. The exact mechanism of inhibition of HC diet-induced LPS production is not clear. It seems that Se decreased LPS release by preventing the lysis of bacterial cell. Uptake of Se might have increased the microbial antioxidant defense system which rendered them to cope with harmful acidic medium. Since the Se supplementation increased the concentration of Se in microflora accompanied with increased glutathione peroxidase (GSH-Px) activity and reduced malondialdehyde (MDA) level [38, 39]. In addition, microbes incorporated and retained much more Se from organic Se compared with inorganic Se compounds [40, 41]. Moreover, Se retention and absorption is higher when supplemented with concentrate diets compared to forage diets [42].

### Epithelial injury, oxidative stress and apoptosis

Concurrent with lowered pH and increased LPS concentrations we found severe damage in colonic mucosa characterized by massive loss of epithelial integrity, depletion of goblet cells and infiltration of inflammatory cells in goats fed HC diet. Several studies reported an increase in serum LPS level in ruminants fed HC diet and suggested that it might be due to increased translocation of LPS from disrupted gut epithelium [43, 44]. In addition, loss of epithelial cells and inflammatory cell infiltration has been reported in rumen, cecum and colon of goats fed high grain diet [10]. However, we observed slight damage to colonic mucosa in goats fed HC diet supplemented with extra Se and no such changes were seen in colon of LC goats. This suggests the attenuating effects of Se against HC diet-induced mucosal injuries. Dietary Se has been shown to exert ameliorative effects on lipid-rich diet-induced aortic injury in rabbit [26] and toxic injury in chicken bursa and jejunum [24]. Moreover, since the Se feeding inhibited LPS production, the minor injury of colonic epithelium in HC-SY group may be due to low luminal pH in colon [45]. This also verifies that low pH together with LPS enhances the extent of injury in rumen and colon tissues [6].

Oxidative stress (OS) is caused either by an increase in pro-oxidants (highly reactive free radicals species) or decrease in the antioxidants, or both [46]. Malondialdehyde (MDA) is as naturally occurring product of lipid peroxidation which reflects the amount of pro-oxidants and thus used as an important OS marker [47]. GSH-Px and SOD are endogenously produced essential components of first-line defense antioxidants which provide protection against reactive oxygen species (ROS). First, the extremely hazardous superoxide anion (O_2_^−^) disumated by SOD into less harmful hydrogen peroxide (H_2_O_2_), which is then broken down to water by GSH-Px. In addition, GSH-Px plays a pivotal role in prevention of oxidative damage by inhibiting lipid peroxidation via converting the lipid peroxides to their corresponding alcohols [48]. Our results showed an increase in MDA content accompanied by decrease in activities of GSH-Px and SOD in colonic epithelium of goats fed HC diet. Concomitant with our results, previous studies have shown that HC diet results in pH reduction and excessive LPS production, thereby develop oxidative stress by increasing MDA content and decreased activities of antioxidant enzymes in gut epithelium and provokes injury [46, 49]. Conversely, extra Se supplementation attenuated HC diet-induced OS in our study. The protective effects of Se on stress mediated mucositis and colitis via OS attenuation has been well documented [50, 51]. Moreover, GSH-Px often known as selenocysteine peroxidase is Se-dependent enzyme and it is believed that its synthesis and secretion is directly related to tissue Se status, which in turn depends upon Se level in diet [52]. In our results, we observed no significant difference in GSH-Px activity between LC and HC, which is due to same background Se level in diet and this also implies that epithelial damage in HC goats is due to excessive free radical formation as evidenced by increased MDA content in HC compared with LC group. On the contrary, extra Se feeding showed 2-fold increase in GSH-Px activity in HC-SY compared to HC group, which might be due to improved tissue Se status and eventually resulted in attenuation of HC diet induced OS and epithelial injury.

HC diet-induced OS triggers cell death in gut epithelium involving apoptotic pathway which impairs the mucosal integrity and eventually results in epithelial leakiness [53, 54]. Apoptosis or programmed cell death is a highly specialized mechanism regulated by a number of specific proteins including bcl2-family and caspases [55]. Bcl2 family contains pro-apoptotic (bax) and anti-apoptotic (Bcl2) proteins that naturally exist in the steady state, and any quantitative change in their expression or fluctuation in the expression of bcl2/bax ratio would alter the apoptotic rate and would ultimately affect the phenotypic status of cell [53]. In our study, concurrent with OS, the HC diet caused cell death in colonic mucosa through apoptosis as evidenced by increase of bax mRNA expression and decrease of bcl2/bax expression ratio compared with LC. Consistently, epithelial disruption with obvious increase in TUNEL-positive apoptotic cells associated with modulation of bcl2 and bax gene expression have been demonstrated in hind gut (Cecum and colon) and rumen of cattle, sheep and goats fed grain rich diets [56–58].

Cell death is induced via caspases-dependent two distinct routes interacting either with the death receptors (The extrinsic pathway) or the mitochondria (The intrinsic pathway) [53]. The bax initiates intrinsic pathway, upon activation by stress signals, it binds to and causes mitochondrial outer membrane permiabilization (MOMP) and releases cytochrome C (cyt, C). By using apoptotic constituent, cyt. C forms complex, the apoptosome which via caspase 9 triggers executioner caspase (Caspase 3) and eventually induces cell death [59]. However, in the extrinsic pathway death receptor upon ligand binding triggers apoptosis via caspase 8. In our study, concomitant with bax, the upregulation of both caspase 3 and caspase 8 demonstrate that HC diet-induced apoptosis in colonic epithelium involves both intrinsic and extrinsic pathways. Similarly, Hua et al. [60] and Tao et al. [54] observed increased mRNA expression levels of bax, caspase 3 and caspase 8 in colonic epithelium of goats fed HC diet in the goats. On contrary, Se supplementation alleviated the HC diet induced apoptotic effects in HC-SY group. However, caspase 8 expression was lower in HC-SY compared to HC goats but it was significantly higher compared to LC which may explain the reason of slight injury of colonic epithelium in HC-SY goats.

## Conclusions

The HC diet-induced OS was associated with apoptosis which led to epithelial injury. Moreover, the over expression of caspase 3 and 8 suggest that HC diet apoptosis involved both intrinsic and extrinsic pathways. However the supranutritional level of Se attenuated the HC diet-induced oxidative stress and apoptosis and thus diminished the epithelial injury. This data for the first time provides the basis for understanding the molecular mechanism underlying the action of Se against HC diet.

## Methods

### Animal selection and feeding management

The experiments performed in this studies were approved by the Institutional Ethical Committe, Sindh Agriculture University Tandojam. Eighteen female cross-bred goats approximately 3–4 months of age, weighing 10–13 kg body weight were randomly selected for study. After the adaptation period of four weeks, animals were moved into individual pens of 2.5 × 4 Sq. ft area per pen. The experimental design was completely randomized. All animals were assigned into three groups (n = 6/group) and fed low concentrate (LC, concentrate: forage; 35: 65), high concentrate (HC, 65:35) or HC plus Se (HC-SY) diets, twice a day at 08:00 and 17:00 h daily with free water access. Selenium was supplemented at the dose rate of 0.5 mg Se kg^− 1^ diet in the form of selenium yeast (Selemax™, Biorigin®, Lençóis Paulista, São Paulo, Brazil). The background Se level in HC and LC diets were 0.15 and 0.035 mg.kg^− 1^ diet, respectively. Selenium at the dose of 0.115 mg.kg^− 1^ diet was added in LC diet to make its concentration equivalent to HC diet and with the supplementation of 0.5 mg Se kg^− 1^, the goats in group HC-SY received total Se by 0.65 mg.kg^− 1^ diet. The chemical composition of diet is given in Table 2. After determining the Se concentration of experimental diets by using inductively coupled plasma-mass spectrometry (ICP-OES Optima 2100-DV, Perkin Elmer) as illustrated by [61] the concentration was adjusted as shown in Table 3. The experimental trial lasted for 10 weeks.

**Table 2:** Composition of diet fed to experimental animals

Items	Treatments	
	**LC**	**HC**
Ingredients (% of DM)		
Corn	25.6	25
Wheat bran	-	30.7
Soybean meal	7.4	2.2
Rapeseed meal	-	4
Lime stone	0.5	1.5
DCP	0.8	0.7
Salt	0.4	0.4
Mineral Premix^1^	0.4	0.4

*DCP *Digestible crude protein.1Per kg of premix= Vitamin A 6 000U; Vitamin D2 500U; Vitamin E 80 mg; Cu 6.25 mg; Fe 62.5 mg; Zn 62.5 mg; Mn 50 mg; I 0.125 mg; Co 0.125 mg; Mo 0.125 mg.

**Table 3:** Selenium level in the diet fed to goats

Se (mg.kg^− 1^ diet)	Treatments		
	**LC**	**HC**	**HC-SY**
Background Se in diet	0.035	0.15	0.15
Added	0.115	-	0.5
Total level	0.15	0.15	0.65

Goats were fed low concentrate (LC), high concentrate (HC) and HC plus selenium (HC-SY) diets for a period of 10 weeks. Selenium was supplemented as selenium yeast (SY) in powder form added to HC diet.

### Slaughter and sample collection

On completion of experimental trial, animals were slaughtered in an isolated slaughter room within animal house so they were not under any pre-slaughter transport stress. After proper restraining in lateral recumbence, the animals were sacrificed by severing the jugular vein, without being stunned, with a razor sharp knife. The slaughter was carried out by a certified and highly skilled technician with a sharp knife. Through abdominal incision, the large intestine was identified, carefully freed from other abdominal viscera and collected into clean tub. Colon was carefully detached; its contents (Digesta) were emptied aseptically into a clean tube and after recording pH, stored at − 20 °C for future analysis. After washing with cold phosphate buffer solution (PBS), three colonic tissue samples were collected: (i) a segment of colonic tissue in 10% formaldehyde for histomorphological evaluation, (ii) epithelial layer was detached through gentle sliding of glass slide on opened colonic wall and collected in empty eppendorf tube and stored frozen until analysis for enzyme activities, and (iii) similarly, some amount of colonic epithelial tissue was preserved for PCR analysis. All analyses were done in blinded fashion.

### Histomorphology and epithelial injury score of colon

For histological evaluations, the formalin fixed tissue samples were dehydrated, cleared and embedded in paraffin. Sections of 4-µm thickness were cut and stained by the standard hematoxylin and eosin (H&E) procedure. Ten replicate measurements for each of the 6 goats per dietary treatment were taken and averaged for statistical analysis. Criteria for histological damaging score was adapted from Tao et al. [10]. Briefly, the severity of epithelial injury was graded as 0–3, from absent to mild (Superficial epithelial injury), moderate (Focal erosions), and severe (Multifocal erosions), the extent of inflammatory cell infiltrate was graded as 0–3, from absent to transmural, and goblet cell depletion was graded as 0–1 present or absent.

### Determination of SCFA and LPS concentration

After adding an equal amount of physiological saline (0.90%w/v of NaCl), the digesta samples were thoroughly mixed and centrifuged (3000 g for 15 min), and then the supernatants were collected into two portions and stored at − 20 °C: First portion was used for SCFA determination as demonstrated by [62] by using capillary column gas chromatography (GC-14B; Shimadzu, Tokyo, Japan; Capillary Column: 30 m × 0.32 mm × 0.25 mm film thickness; column temperature 110 °C; injector temperature 180 °C; and detector temperature 180 °C). Another portion was used for LPS detection by chromogenic End-point Tachypleus Amebocyte Lysate Assay Kit (Chinese Horseshoe Crab Reagent Manufactory Co. Ltd, Xiamen China). Pretreated supernatants were diluted until LPS concentrations were in the range of 0.1–1.0 endotoxin unit ml^− 1^ comparative to the reference endotoxin and analyzed as described by [4].

### MDA level and anti-oxidant activity assay

The colonic epithelial tissue lysates were prepared by homogenization in standardized PBS buffer. The MDA content and activities of GSH-Px, SOD and CAT were measured by using ELISA kits (Nanjing Jincheng Bioengineering Institute China). Standard protocols and procedures provided by the manufacturer were followed.

### Total RNA isolation, real-time Rt-PCR

Total RNA was extracted from colonic tissue samples by using acid guanidinium thiocyanate-phenol-chloroform (GTC) method [7]. The RNA concentration was measured at 260 and 280 nm by using nano drop spectrometer. All samples had an absorbance ratio between of 1.72 and 1.84 indicating high RNA purity. Real time PCR was carried out in a total volume of 20 ul containing 1xiQ SYBR Green supermix (Bio-Rad Laboratories, Inc., Hercules, CA), a mixture of forward and reverse primers (Table 4), cDNA template and a known amount of sterile water. An initial cycle of 30 s at 95 °C was used to denature the cDNA. This was followed with 40 PCR cycles consisting of denaturation at 95 °C for 10 sec and primer annealing and extension at 55 °C for 30 sec. Before performing the PCRs for experimental samples, amplification efficiencies of all primers by using standard dilution series was calculated. After PCR analysis a melt analysis was carried out, all samples were analyzed in triplicate. Gene expression was normalized to GAPDH (∆Ct = Ct _target_ – Ct _GAPDH_). The relative expression values were calculated using the formula 2^−∆∆Ct^ as described by [63].

**Table 4:** Primers for real time RT-PCR

Gene	Primer sequence 5’ to 3’	Accession number	Size(bp)
GAPDH	GGGTCATCATCTCTGCACCT	HM043737.1	180
	GGTCATAAGTCCCTCCACGA		
Bcl2	TCGCCCAAGTCAAACATTA	AY423861.1	208
	CACAGGTGAAACTGCCAAGAT		
Bax	TGCTCACTGCCTCACTCAC	AF163774.1	178
	CCAAGACCACTCCTCCCTA		
Caspase3	GGTTCATCCAGGCTCTTT	AF068837.1	98
	TTCTGTCGCTACCTTTCG		
Caspase8	GGCTCCTCTGAGATGCTG	NM-001045970	149
	TGCTCCCGTGCTATGCTA		

GAPDH mRNA, Glyceraldehyde 3-phosphate dehydrogenase ribosomal RNA; Bcl-2, B-cell lymphoma 2; Bax, Bcl-2-associated x protein; Caspase, cysteine-aspartic proteases. The first primer listed for each gene is the forward primer and the second primer is reverse primer

### Statistical Analysis

The data was analyzed through statistical program by using SPSS 16.0 (Stata Soft, Tulsa, OK, USA) and assessed by a one-way ANOVA. Data is expressed as the means ± standard error. Differences with a *P*-value of < 0.05 were considered significant.

## Data Availability

The datasets used and analyzed during current study can be provided by corresponding author on practical request.

## Abbreviations

HC: High concentrate
LPS: Lipopolysaccharide
OS: Oxidative stress
ROS: Reactive oxygen species
H_2_O_2_: Hydrogen peroxide
Se: Selenium
SY: Selenium yeast
DM: Dry matter
MDA: Melondialdehyde
GSH-Px: Glutathione peroxidase
SOD: Super oxide dismutase
CAT: Catalase
GADPH: Gluteraldehyde phosphate dehydrogenase
SCFA: Short chain fatty acid
MOMP: Mitochondrial outer membrane permiabilization
ELISA: Enzyme-linked immunosorbent assay
TUNEL: Terminal deoxynucleotidyl transferase dUTP nick end labeling
